# Supplementary material for: Computational Model Reveals a Stochastic Mechanism behind Germinal Center Clonal Bursts
Source: Cells. 2020 Jun 10;9(6):1448. doi: 10.3390/cells9061448 (PMC7349200; doi:10.3390/cells9061448)
Supplement: Supplementary file 1 [file cells-09-01448-s001.pdf]

cells

MDPI

Article

Supplementary information of: Computational model reveals a stochastic mechanism behind GC clonal bursts

Aurélien Pélissier [1,2,*], Youcef Akrout [3], Katharina Jahn[2], Jack Kuiper[2], Ulf Klein[4], Niko Beerenwinkel[2] and María Rodríguez Martínez [1,*]

1 IBM Research Zurich, 8803 Rüschlikon, Switzerland
2 Department of Biosystems Science and Engineering, ETH Zurich, Basel, Switzerland; katharina.jahn@bsse.ethz.ch (K.J.); jack.kuipers@bsse.ethz.ch (J.K.); niko.beerenwinkel@bsse.ethz.ch (N.B.)
3 École Normale Supérieure, Paris, France; youcef.akrout@ens.fr
4 Leeds Institute of Medical Research at St. James's, University of Leeds, Leeds, UK; u.p.klein@leeds.ac.uk
* Correspondence: pel@zurich.ibm.com (A.P.); mrm@zurich.ibm.com (M.R.M.)

1. A simple GC kinetics model with ODEs

By assuming idealized communication between cells in the lymph node, we can model the germinal center (GC) dynamics using a system of Ordinary Differential Equations (ODE) [1]. However, some adjustments are needed to correctly reproduce the complex model described in the main document. Let $\delta_{\text{div}} < 1$ be the probability of a centroblast's daughter undergoing apoptosis after a cellular division. Let n_{div} be the average number of divisions undergone by centroblasts before migrating to the LZ.

First, since one division produces 2 daughter cells, $2\delta_{\text{div}}$ cells die on average after each division event. Second, since the number of division per centroblast is limited to an average of n_{div} divisions per DZ–LZ cycle, we need to distinguish centroblasts that are actively undergoing division, CB1, versus centroblasts that have finalised replication (their division counter has dropped to 0) and are in the process of migrating to the LZ, CB0. Thus, we consider that a replicating cell will become a migrating centroblast with probability m after each division event. Using the same notation described in the main text, the ODE system governing GC kinetics can be written as follows:

$$\begin{aligned}
\frac{dN_{\text{CB1}}}{dt} &= r_{\text{recirculate}} \cdot N_{\text{CCsel}} + r_{\text{div}} \left[(1-m)(2-2\delta_{\text{division}}) - 1 \right] N_{\text{CB1}} \\
\frac{dN_{\text{CB0}}}{dt} &= r_{\text{div}} \cdot m(2-2\delta_{\text{division}}) N_{\text{CB1}} - r_{\text{migration}} \cdot N_{\text{CB0}} \\
\frac{dN_{\text{CC}}}{dt} &= r_{\text{migration}} \cdot N_{\text{CB0}} - r_{\text{apoptosis}} \cdot N_{\text{CC}} - r_{\text{TC:CC}} \cdot (N_{\text{T}_{\text{FH}}})_{\text{unbound}} \\
\frac{dN_{\text{CCTC}}}{dt} &= r_{\text{TC:CC}} \cdot (N_{\text{T}_{\text{FH}}})_{\text{unbound}} - r_{\text{unbinding}} \cdot N_{\text{CCTC}} \\
\frac{dN_{\text{CCsel}}}{dt} &= r_{\text{unbinding}} \cdot N_{\text{CCTC}} - r_{\text{exit}} \cdot N_{\text{CCsel}} - r_{\text{recirculate}} \cdot N_{\text{CCsel}}
\end{aligned} \tag{1}$$

The number of centroblasts in the main text, N_{CB}, is equivalent to $N_{\text{CB1}} + N_{\text{CB0}}$ in the ODE model. Likewise, δ_{div} is related to δ, the probability of dying after SHM defined in the main text, according to $\delta_{\text{div}} = \delta \cdot N_{\text{BCR}} \cdot p_{\text{SHM}}$, where $N_{\text{BCR}} \cdot p_{\text{SHM}} = 0.66$ is the average number of mutations that occur on each replication (see Section 2.2 in the main text). m has been estimated numerically to replicate the results of the stochastic simulation, with an optimal value of $m = 0.378$. In this simplified model, we only consider the spontaneous unbinding of a CC, which leads to its activation. We note that the unbinding as a result of CC competition only leads to the replacement of two CCs, but leaves the total

population counts unchanged. Finally, the number of bounded centrocytes N_{CCTC} can be assumed to be roughly equal to the total number of $(N_{\mathrm{T_{FH}}})_{\mathrm{total}} = \alpha_{\mathrm{TC}} \cdot N_{\mathrm{CC}}$ – similarly to the main simulation, the T_{FH} encounter rate is large, which ensures that ∼99 % of the T_{FHs} are bonded at all times.

This ODE system is a powerful tool to constrain our parameters because solving an ODE system numerically is orders of magnitude faster than running the Gillespie algorithm. Note that however, this model is limited, as it does not provide any insight about affinity maturation, SHM events, and clonal diversity.

2. Constraining parameters from literature

We describe next the derivation of stochastic model parameters from the literature and the variability bounds associated with each one.

- **Affinity of GC founder cells**: The initial activation of seeder cells by T_{FHs} is competitive, and only the highest-affinity naive B cells are selected to enter the GC [2]. By selecting the top 1% from random sequences, we obtain an estimate of an average founder affinity of ∼0.4 (the affinity of a random BCR is 0.25 on average). All founders are set to an equal affinity to ensure a fair clonal competition at the beginning of the GC response.

- $r_{\mathbf{activation}}$: Little is known about how fast founder cells enter the GC reaction, which might vary through time. We thus set a wide range of variability for $r_{\mathrm{activation}}$ of 1-10 cells/h.

- $r_{\mathbf{division}}$: GC B cells are among the fastest dividing mammalian cells, with a cell-cycle lasting between 6-12 h [3]. We assume that one division has occurred after a cell cycle is completed. In addition, the Gillespie algorithm assumes constant reaction rates, hence, the time between two subsequent events follows an exponential distribution [4] with rate parameter $\lambda = r_{\mathrm{division}}$, i.e. $\mathrm{P}_{\mathrm{division}} = \lambda \cdot \exp(-\lambda \cdot x)$. The mean waiting time between two centroblast divisions can be estimated as the mean value of $\mathrm{P}_{\mathrm{division}}$, i.e.:

$$< \mathrm{t}_{\mathrm{division}} >= \int_0^\infty \lambda \cdot x \cdot \exp(-\lambda \cdot x)\, dx = \frac{1}{\lambda} = \frac{1}{r_{\mathrm{division}}} = 6 - 12\,\mathrm{h}\,,$$

  from where $\mathrm{r}_{\mathrm{division}} = 0.08$ - 0.16/h.

- $r_{\mathbf{recirculate}}$: Assuming that a selected centrocyte diffuses back into the dark zone at speed $v_{\mathrm{CC}} = 5\,\mu\mathrm{m/min}$, recirculation will take $t_{\mathrm{recirculate}} \simeq \frac{r_{\mathrm{GC}}}{v_{\mathrm{CC}}} = \frac{80\,\mu\mathrm{m}}{5\,\mu\mathrm{m/min}} = 16\,\mathrm{min}$, which suggests $r_{\mathrm{recirculate}} = \frac{1}{t_{\mathrm{recirculate}}} = 3.75/\mathrm{h}$

- $r_{\mathbf{exit}}$: Assuming that both the recirculation and GC exit are independent random variables drawn from an exponential distribution, then:

$$P(t_{\mathrm{recirculate}} < t_{\mathrm{exit}}) = \frac{r_{\mathrm{recirculate}}}{r_{\mathrm{recirculate}} + r_{\mathrm{exit}}}\,.$$

  After being rescued by a T_{FH}, a centrocyte will either recirculate in the DZ ($r_{\mathrm{recirculate}}$) or exit the GC (r_{exit}). The fraction of selected centrocytes that recirculate has not been accurately determine, and models suggest that the majority of centrocytes should recirculate, as it leads to a more efficient affinity maturation [5]. Therefore, we consider a broad range of variability for the recirculation fraction between 50-90 %, which leads to $r_{\mathrm{exit}} \sim 0.42$-3.75/h.

- $r_{\mathbf{migration}}$: In vivo experiments [6] reported that 15% of centroblasts migrate to the LZ every hour, leading to a migration rate of ∼ 0.15/h. However, this rate is defined relatively to the total number of centroblasts. In our model, only centroblasts with divisions counters equal to 0 are allowed to migrate, and hence, we need to increase $r_{\mathrm{migration}}$ to ∼ 3/h to match the experimental

observations [6]. Modeling a migration as a diffusion process at speed $v_{\mathrm{CC}} = 5\,\mu\mathrm{m/min}$ for a GC radius of 80 µm leads to an upper bound of 3.75/h.

- $r_{\mathbf{apoptosis}}$: The typical life time of centrocytes has been estimated to be 6 to 16 hours [7], which leads to $r_{\mathrm{apoptosis}}$ ranging between 0.06 - 0.17/h.

- δ: The probability of nonproductive mutations leading to apoptosis is difficult to infer from repertoire sequencing data, as only the surviving BCR sequences are measured. Thus, we set a broad range of variability for this parameter, $\delta \sim 0.1 - 0.9$.

- $\mathbf{pMHC_{threshold}}$: This parameter arises as a hypothesis in our model, which has never been described experimentally. Hence, we leave this parameter unconstrained, i.e. ranging between 0 and 1.

- $p_{\mathbf{SHM}}$: On average, the mutation rate per site in the variable region has been determined to be $p_{\mathrm{SHM}} = 1 \times 10^{-3}$ [8].

- $N_{\mathbf{FDC}}$: An average of 250 FDCs were found experimentally in mature GCs [9].

- $\alpha_{\mathbf{TC}}$: [10] reported a TC:CC ratio of 1/7 in the light zone. However, as T_{FHs} are specialized to different types of antigens, and as we only consider one antigen in our simulation, we set the lower bound to 1/100 during the optimisation of α_{TC}.

- $r_{\mathbf{TC:CC}}$: The rate of encounter with T_{FHs} for a single centrocyte can be approximated by the volume swept by the CC (relative to the T_{FHs}) per unit time multiplied by the density of unbounded T_{FHs} in the light zone:

$$r_0 \approx (v_{\mathrm{CC}} + v_{\mathrm{TC}}) \cdot A_{\mathrm{effect}} \cdot \frac{(N_{\mathrm{T_{FH}}})_{\mathrm{unbound}}}{\mathrm{Vol_{LZ}}}\,, \tag{2}$$

where A_{effect} is the effective area of interaction of a centrocyte, and v_{CC} and v_{TC}, the diffusion rates of centrocytes and T_{FHs} respectively. Hence, the propensity of encounters of CCs and T_{FHs} can be written as follows:

$$\begin{aligned}
&\text{propensity of encounters TC:CC} = \\
=&(\text{volume swept by a CC relative to a T cell per unit time}) \cdot (\text{density of unbounded T cells}) \cdot \\
&\cdot (\text{number of CCs}) \\
=&((v_{\mathrm{CC}} + v_{\mathrm{TC}})\pi r_B^2) \cdot (\frac{(N_{\mathrm{T_{FH}}})_{\mathrm{unbound}}}{\mathrm{Vol_{LZ}}}) \cdot N_{\mathrm{CC}} \\
\simeq&((v_{\mathrm{CC}} + v_{\mathrm{TC}})\pi r_B^2) \cdot (\frac{(N_{\mathrm{T_{FH}}})_{\mathrm{unbound}}}{\mathrm{Vol_{B\,cell}} \cdot N_{\mathrm{CC}}}) \cdot N_{\mathrm{CC}} \\
=&((v_{\mathrm{CC}} + v_{\mathrm{TC}})\pi r_B^2) \cdot (\frac{(N_{\mathrm{T_{FH}}})_{\mathrm{unbound}}}{\frac{4}{3}\pi r_B^3}) \\
=&\frac{3}{4r_B}(v_{\mathrm{CC}} + v_{\mathrm{TC}}) \cdot (N_{\mathrm{T_{FH}}})_{\mathrm{unbound}} = r_{\mathrm{TC:CC}} \cdot (N_{\mathrm{T_{FH}}})_{\mathrm{unbound}}\,.
\end{aligned}$$

In the above derivation we have assumed that the reaction volume is proportional to the number of GC B cells, thus we can write $\mathrm{Vol_{LZ}} = V_{\mathrm{B\,cell}} \cdot N_{\mathrm{CC}}$. Taking the typical cell velocities obtained from two-photon microscopy experiments [11], $v_{\mathrm{TC}} = 15\,\mu\mathrm{m/min}$ and $v_{\mathrm{BC}} = 5\,\mu\mathrm{m/min}$, and a typical B cell and T_{FH} radius of 6.2 µm, we obtain $r_{\mathrm{TC:CC}} = \frac{145}{N_{\mathrm{CC}}}/\mathrm{h}$, which is consistent with the finding that a GC B cell can encounter as many as 50 T cells per hour [11].

The calculation presented here is approximate, because the real motion of T_{FHs} and CC is in general Brownian. Hence, the volume swept by a CC per unit time can be expected to be smaller than the volume obtained by the linear addition of v_{CC} and v_{TC}. However, the derivation gives a correct order of magnitude.

- $r_{\mathbf{FDC:CC}}$: We follow an analogous reasoning to the encounter of T_{FHs}, and estimate the propensity of encounters between a centrocyte and an FDC by assuming that FDCs do not diffuse, i.e. $v_{FDC} = 0$. With this assumption, we obtain $r_{FDC:CC} = \frac{40}{N_{CC}}/h$. Note that this rate is orders of magnitude lower than the typical rate of an FDC-CC interaction, for which the length is of the order of ~ 1 second.

- $r_{\mathbf{unbinding}}$: In vivo studies suggest that B cells integrate signals from many short contacts with T_{FHs} of less than 5 minutes each [11]. However, some B cells can engage in longer contacts with T_{FHs} for up to 30 min, leading to a spontaneous unbinding rate $r_{unbinding} = \frac{1}{30\text{ min}} = 2/h$.

- $N_{\mathbf{CDR}}$: The nucleotide length of the CDR only influences the computation of the affinity values (by changing the discretized units of affinity that a receptor can gain or loses after each mutation event), however it does not result in quantitative changes in the measurable GC output. Since GC B cells typically acquire up to 20 mutations on their BCRs throughout the GC lifespan [12], most of which occur in the CDR region, we set N_{CDR} to 25.

- σ: We assume a rate of neutral mutations of $\sim$0.28, as suggested in the mutation fate tree provided in [13].

3. Details about optimisation with `maxLIPO`

3.1. *A global optimization algorithm*

`LIPO` is a state of the art global optimization algorithm that leads to efficient optimization of an unknown function f under the only assumption that it is continuous [14]. It is both parameter free and provably better than random search. However, while the LIPO exploration procedure quickly get onto the global optima area (i.e. finding the highest peak), it does not make very rapid progress towards the optimal location (i.e. the very top of the peak). To improve performance in optimizations, `LIPO` is typically combined with *classic trust region* methods such as `BOBYQA` [15]. In our work, the `[LIPO + BOBYQA]` implementation from the `dlib` library, referred to as `maxLIPO`, was used [16]. `maxLIPO` is a good alternative to Bayesian optimization methods [17], which typically require the selection of a prior that expresses assumptions about the function being optimized, and thus require domain knowledge. Bayesian methods may lead to poor performance if the priors are not cautiously chosen.

3.2. *The score function*

Let $M(x) = (M_1..M_n)(x)$ be the output of our model for a set of input parameters $x \in \mathbb{R}^d$, and let $E = (E_1..E_n)$ be the experimental data that our model aims to replicate. We define a score function $f : \mathbb{R}^d \rightarrow \mathbb{R}$ to quantify how our model performs compared to experimental data. $f(x)$ is defined as the mean squared error (MSE) between experiments and model predictions:

$$f(x) = \sum_{i=1}^{n} k_i (M_i(x) - E_i)^2 . \quad (3)$$

The coefficient k_i is used to tune the *importance* of a given output value relatively to other values. The value k_i was defined as the inverse of the variance of experimental data, thus ensuring a fair weight

between different GC measured properties. If a simulation M diverges, we set the score function to be equal to that obtained from an empty GC, which results in a bad score for that simulation.

3.3. Optimization results

The algorithm `maxLIPO` typically requires at least thousands of iterations to find the global optimum of the score function. It is therefore crucial to have a score that can be quickly computed to run the algorithm in a reasonable time. To shorten the optimisation to feasible running times, we use the deterministic ODE system described by Eqs. 1 instead of the full Gillespie simulation.

After initializing the ODE system with 1000 centroblasts and 1000 centrocytes, the parameters r_{division}, $r_{\text{migration}}$, $r_{\text{apoptosis}}$, r_{exit}, α_{TC} and δ were optimized with `maxLIPO` using the GC kinetics data described in section 2.10 of the main text. Note that $n_{\text{div}} = 3.5$ is fixed by the definition of our Myc-driven division model based on 6 percentiles. We found that running `maxLIPO` with 3000 iterations was enough to obtain a good optimum, i.e. the score did not improve for more than 300 iterations. Once these parameters were constrained, other parameters were adjusted with the main Gillespie simulation as follows:

- $r_{\text{activation}}$ was fitted such that the GC peaks at 2000 cells at day 9. Note that this number depends on the lethal mutation probability δ, as more founders are required to reach the peak if a higher number of cells die after proliferation.
- $\text{pMHC}_{\text{threshold}}$, and additional parameters used in to test the different differentiation scenarios (section 2.7, in the main text): These parameters only affect the productiong of MBCs and PCs, and they were fixed to minimize the average NRMSD of both PC and MBC production.

3.4. T_{FH} surviving signal

The T_{FH} surviving signal in section 2.5 of the main text is defined as:

$$\text{signal strength (pMHC)} = \exp(\text{pMHC}^n) - 1\,. \tag{4}$$

In our model, the amount of received T_{FH} signals determines the number of cellular divisions a centroblast undergoes in the dark zone by setting the value of the division counter (section 2.6 in the main text). Setting the division counter according to the amount of received T_{FH} signals defined by Eq. 4 leads to GC dynamics incompatible with observations if n is kept constant (Supplementary figure S1). For instance, for n constant, Supplementary figure S1A shows that the volume of a GC decreases around day 20 only to increase again at late time points. This anomalous behaviour can be understood if one considers that late GC B cells have higher affinity, therefore they acquire T_{FH} signals more easily (according to Eq. 4) and divide more often than early GC B cells (Supplementary figures S1A and S1C). Supporting this intuition, centroblasts divide on average 4.5 times at day 40, in comparison to an average of 3.5 divisions expected from our 6 percentiles model (Supplementary figure S1C). Thus, to ensure that the average amount of received T_{FH} signals remains roughly constant through the GC reaction, we assume that the parameter n increases with time as follows:

$$< \text{pMHC(t)} >^{n(t)} \simeq < \text{pMHC}(\text{t}_0) >^{n(t_0)}, \tag{5}$$

where < pMHC(t)> refers to the average pMHC of all GC B cells at time t and t_0 is the starting time of the GC reaction (day 4). This equation ensures that the T_{FH} distribution is consistent at all time points, and that the GC behaves as expected with a consistent average of 3.5 divisions per centroblast, as seen on Supplementary figures S1B and S1D.

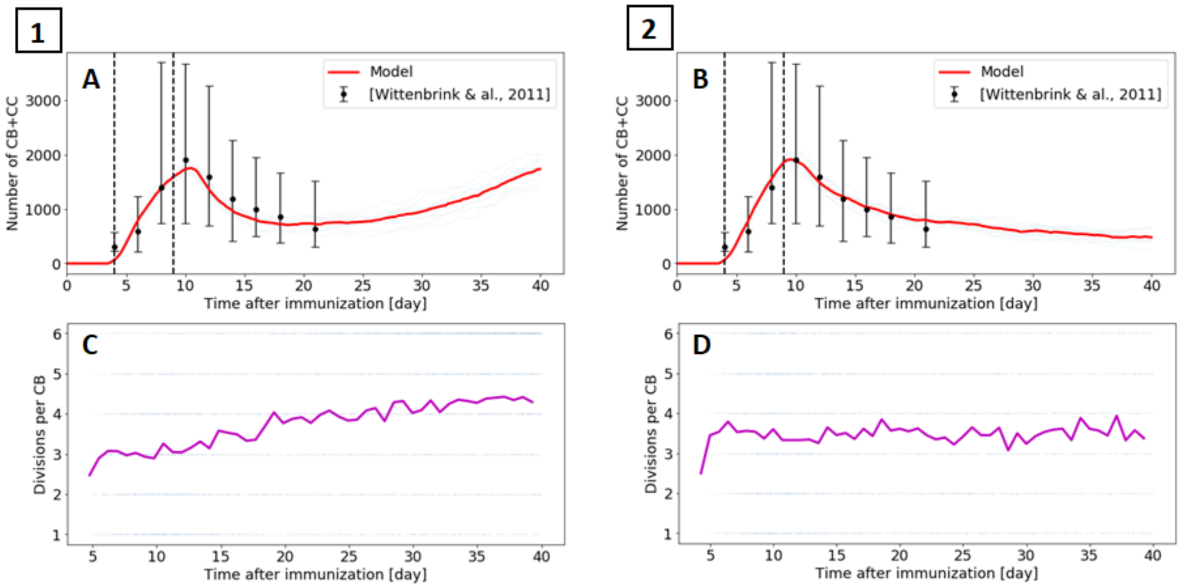

Supplementary Figure S1. (A,C) Germinal center B cell count and (B,D) average number of divisions per centroblast as a function of time for i) $n = 2$ in Eq. 4 and ii) dynamic n adjusted from Eq 5.

4. Sensitivity analysis of the agent based model

A sensitivity analysis was performed in which individual parameters were changed successively by ±10 %. The changes in the model output were then quantified by the change in the average NRMSD over all GC characteristics (Supplementary Table S1), computed as:

$$sensitivity = \frac{< \text{new NRMSD} > - < \text{old NRMSD} >}{< \text{old NRMSD} >} \quad (6)$$

Changing the parameters had various effects:

- The GC half life (amount of time it takes for the GC to reduce its size by half) is the result of a tightly regulated balance between apoptosis ($r_{\text{apoptosis}}$, δ, p_{SHM}, $r_{\text{apoptosis}}$), exit (r_{exit}, $r_{\text{recirculate}}$) and the positive selection by T_{FH} ($r_{\text{unbinding}}$, α_{TC}). Any small change in one of these parameters significantly changes the GC decay dynamics, and thus the GC half life. Some parameter changes resulted in a diverging GC (GC undergoing infinite growth rather than decay), and thus a significantly higher RMSD than when the GC is shutting down.
- $r_{\text{activation}}$ controls the rate at which cells enter the GC, and a faster rate leads to a higher GC B cell count at day 9. A high variance in the activation rate could be a main factor to explain the a high variability in GC sizes observed experimentally [10]. We note however that despite GC size variability,the GC half life seems to be relatively insensitive to $r_{\text{activation}}$.
- r_{division} and $r_{\text{migration}}$ control the time centroblasts spent in the DZ. Shorter times in the DZ relative to the LZ reduces the DZ/LZ ratio.
- $r_{\text{TC:CC}}$, $r_{\text{FDC:CC}}$ and N_{FDC} account for the competition between B cells. Less interactions between FDCs and centrocytes result in longer times needed to acquire antigen, and thus, less time remaining to obtain T_{FH} help. On the other hand, more frequent interactions between T_{FH} and centrocytes increases the B cell competition, as it is more likely for a B cell to be displaced of a T_{FH} by a B cell exhibiting a higher affinity BCR.
- $\text{pMHC}_{\text{threshold}}$, and its equivalent in the other models, is the only parameter that controls the ratio of PC versus MBC production.

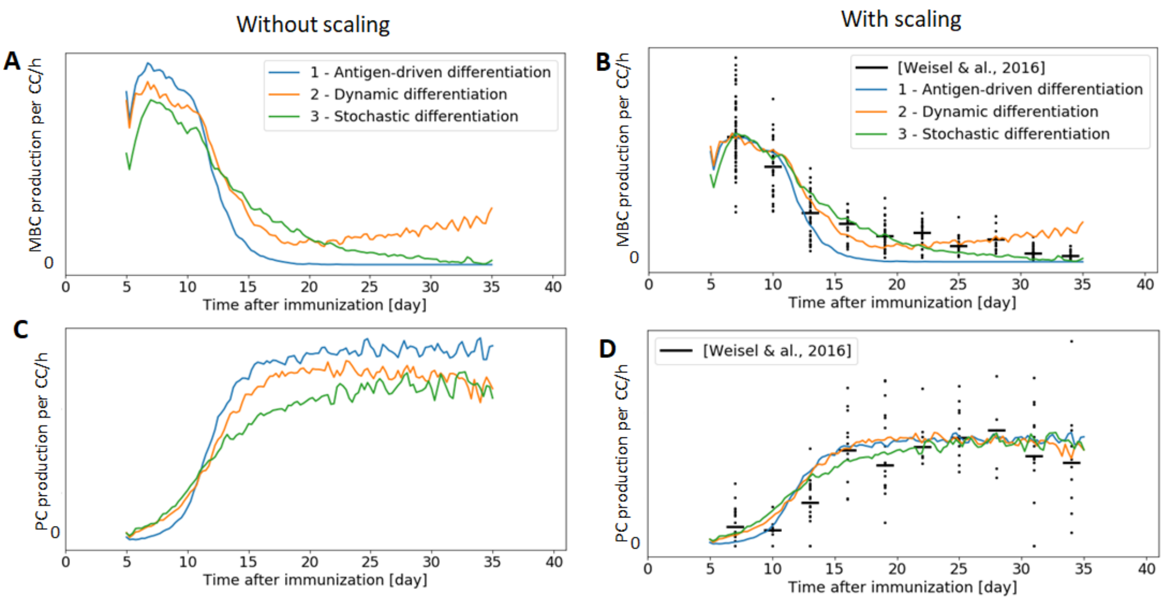

Supplementary Figure S2. Memory cells (A,B) and plasma cells (C,D) production with and without scaling for the 3 B cell differentiation models described in the main text section 2.7. To compare with experimental values, the model predictions and experimental measures were scaled by their maximum values, implying a loss of information about the absolute production amount of MBC and PC.

5. Supplementary figures

1. Oprea, M.; Perelson, A.S. Somatic mutation leads to efficient affinity maturation when centrocytes recycle back to centroblasts. *The Journal of Immunology* **1997**, *158*, 5155–5162.
2. Mesin, L.; Ersching, J.; Victora, G.D. Germinal center B cell dynamics. *Immunity* **2016**, *45*, 471–482.
3. Victora, G.D.; Nussenzweig, M.C. Germinal centers. *Annual Review of Immunology* **2012**, *30*, 429–457. doi:10.1146/annurev-immunol-020711-075032.
4. Thomas, M.J.; Klein, U.; Lygeros, J.; Rodríguez Martínez, M. A probabilistic model of the germinal center reaction. *Frontiers in immunology* **2019**, *10*, 689.
5. Meyer-Hermann, M.; Mohr, E.; Pelletier, N.; Zhang, Y.; Victora, G.D.; Toellner, K.M. A theory of germinal center B cell selection, division, and exit. *Cell reports* **2012**, *2*, 162–174.
6. Victora, G.D.; Schwickert, T.A.; Fooksman, D.R.; Kamphorst, A.O.; Meyer-Hermann, M.; Dustin, M.L.; Nussenzweig, M.C. Germinal center dynamics revealed by multiphoton microscopy with a photoactivatable fluorescent reporter. *Cell* **2010**, *143*, 592–605.
7. Liu, Y.J.; Barthelemy, C.; de Bouteiller, O.; Banchereau, J. The differences in survival and phenotype between centroblasts and centrocytes. In *In Vivo Immunology*; Springer, 1994; pp. 213–218.
8. Kleinstein, S.H.; Louzoun, Y.; Shlomchik, M.J. Estimating hypermutation rates from clonal tree data. *The Journal of Immunology* **2003**, *171*, 4639–4649.
9. Wittenbrink, N.; Weber, T.S.; Klein, A.; Weiser, A.A.; Zuschratter, W.; Sibila, M.; Schuchhardt, J.; Or-Guil, M. Broad volume distributions indicate nonsynchronized growth and suggest sudden collapses of germinal center B cell populations. *The journal of immunology* **2010**, *184*, 1339–1347.
10. Wittenbrink, N.; Klein, A.; Weiser, A.A.; Schuchhardt, J.; Or-Guil, M. Is there a typical germinal center? A large-scale immunohistological study on the cellular composition of germinal centers during the hapten-carrier–driven primary immune response in mice. *The Journal of Immunology* **2011**, *187*, 6185–6196.
11. Allen, C.D.; Okada, T.; Tang, H.L.; Cyster, J.G. Imaging of germinal center selection events during affinity maturation. *Science* **2007**, *315*, 528–531.

12. Abbott, R.K.; Lee, J.H.; Menis, S.; Skog, P.; Rossi, M.; Ota, T.; Kulp, D.W.; Bhullar, D.; Kalyuzhniy, O.; Havenar-Daughton, C.; others. Precursor frequency and affinity determine B cell competitive fitness in germinal centers, tested with germline-targeting HIV vaccine immunogens. *Immunity* **2018**, *48*, 133–146.
13. Reshetova, P.; van Schaik, B.D.; Klarenbeek, P.L.; Doorenspleet, M.E.; Esveldt, R.E.; Tak, P.P.; Guikema, J.E.; de Vries, N.; van Kampen, A.H. Computational model reveals limited correlation between germinal center B-cell subclone abundancy and affinity: implications for repertoire sequencing. *Frontiers in immunology* **2017**, *8*, 221.
14. Malherbe, C.; Vayatis, N. Global optimization of lipschitz functions. Proceedings of the 34th International Conference on Machine Learning-Volume 70. JMLR. org, 2017, pp. 2314–2323.
15. Powell, M.J. The BOBYQA algorithm for bound constrained optimization without derivatives. *Cambridge NA Report NA2009/06, University of Cambridge, Cambridge* **2009**, pp. 26–46.
16. Davis, K. A Global Optimization Algorithm Worth Using. dlib C++ library, 2017.
17. Snoek, J.; Larochelle, H.; Adams, R.P. Practical bayesian optimization of machine learning algorithms. Advances in neural information processing systems, 2012, pp. 2951–2959.

© 2020 by the authors. Licensee MDPI, Basel, Switzerland. This article is an open access article distributed under the terms and conditions of the Creative Commons Attribution (CC BY) license (http://creativecommons.org/licenses/by/4.0/).

Supplementary Table S1. Sensitivity analysis of all the parameters used in our model. The model sensitivity is quantified by the change in the average NRMSE over all GC characteristic, averaged over 100 simulations. Due to the stochastic nature of the Gillespie algorithm, the sensitivity is provided within a confidence range of $\pm 10\,\%$
.

Parameter	Original value	Value change	Model sensitivity	Main effect
$r_{\text{activation}}$ [h^{-1}]	3.94	$-10\,\%$	$127\,\%$	Value of GC peak
		$+10\,\%$	$82\,\%$	
r_{division} [h^{-1}]	0.134	$-10\,\%$	$54\,\%$	GC decay and DZ/LZ ratio
		$+10\,\%$	$27\,\%$	
$r_{\text{migration}}$ [h^{-1}]	3.75	$-10\,\%$	$< 10\,\%$	DZ/LZ ratio
		$+10\,\%$	$< 10\,\%$	
$r_{\text{apoptosis}}$ [h^{-1}]	0.084	$-10\,\%$	$715\,\%$	GC decay and CC apoptosis
		$+10\,\%$	$126\,\%$	
r_{exit} [h^{-1}]	1.64	$-10\,\%$	$324\,\%$	GC decay and CC apoptosis
		$+10\,\%$	$86\,\%$	
$r_{\text{recirculate}}$ [h^{-1}]	3.75	$-10\,\%$	$103\,\%$	GC decay and CC apoptosis
		$+10\,\%$	$347\,\%$	
$r_{\text{FDC:CC}}$ [h^{-1}]	$\frac{40}{N_{\text{CC}}}$	$-10\,\%$	$< 10\,\%$	Clonal competition
		$+10\,\%$	$< 10\,\%$	
$r_{\text{TC:CC}}$ [h^{-1}]	$\frac{145}{N_{\text{CC}}}$	$-10\,\%$	$< 10\,\%$	Clonal competition
		$+10\,\%$	$29\,\%$	
$r_{\text{unbinding}}$ [h^{-1}]	2	$-10\,\%$	$97\,\%$	GC decay
		$+10\,\%$	$587\,\%$	
$\alpha_{\text{TC}} = N_{\text{TC}}/N_{\text{CC}}$	1/46	$-10\,\%$	$97\,\%$	GC decay
		$+10\,\%$	$587\,\%$	
N_{FDC}	250	$-10\,\%$	$< 10\,\%$	Clonal competition
		$+10\,\%$	$< 10\,\%$	
p_{SHM}	1×10^{-3}	$-10\,\%$	$690\,\%$	GC decay and CB apoptosis
		$+10\,\%$	$110\,\%$	
δ	0.52	$-10\,\%$	$690\,\%$	GC decay and CB apoptosis
		$+10\,\%$	$110\,\%$	
σ	0.28	$-10\,\%$	$< 10\,\%$	Affinity maturation
		$+10\,\%$	$< 10\,\%$	
N_{site}	25	$-10\,\%$	$< 10\,\%$	Affinity maturation
		$+10\,\%$	$< 10\,\%$	
$\text{pMHC}_{\text{threshold}}$	0.46	$-10\,\%$	$36\,\%$	PC/MBC production
		$+10\,\%$	$17\,\%$	
